# The Mechanism and Effect of Autophagy, Apoptosis, and Pyroptosis on the Progression of Silicosis

**DOI:** 10.3390/ijms22158110

**Published:** 2021-07-28

**Authors:** Shiyi Tan, Shi Chen

**Affiliations:** Key Laboratory of Molecular Epidemiology of Hunan Province, Hunan Normal University, Changsha 410013, China; eleven@hunnu.edu.cn

**Keywords:** programmed cell death, autophagy, apoptosis, pyroptosis, silicosis

## Abstract

Silicosis remains one of the most severe pulmonary fibrotic diseases worldwide, caused by chronic exposure to silica dust. In this review, we have proposed that programmed cell death (PCD), including autophagy, apoptosis, and pyroptosis, is closely associated with silicosis progression. Furthermore, some autophagy, apoptosis, or pyroptosis-related signaling pathways or regulatory proteins have also been summarized to contribute greatly to the formation and development of silicosis. In addition, silicosis pathogenesis depends on the crosstalk among these three ways of PCD to a certain extent. In summary, more profound research on these mechanisms and effects may be expected to become promising targets for intervention or therapeutic methods of silicosis in the future.

## 1. Programmed Cell Death Is Necessary for Participation in the Regulatory Mechanism of Silicosis

Silicosis is a devastating interstitial lung disease characterized by silicon nodules and diffuse pulmonary fibrosis. It is a severe occupational hazard disease worldwide caused by long-term inhalation of crystalline-free silica dust in the workplaces (referred to as silica after this) [1,2]. Programmed cell death (PCD) refers to an active cell death process to maintain the internal environment’s stability after receiving a specific signal or stimulating factors [3]. Typical forms of PCD include autophagy, apoptosis, or pyrolysis, etc. [4]. Currently, increasing evidence has shown that PCD performs a necessary role in the pathogenesis of silicosis.

### 1.1. Silicosis Is a Complex Occupational Hazard Disease Worldwide

The current widely accepted silicosis pathogenesis is as follows: (1) Silica is identified and then phagocytosed by the alveolar macrophage (AM) via the scavenger receptor, which is the first critical defensive line for silica invasion [5,6]. Silicosis is developed through a vicious circle. AM engulfs silica to cause AM death and then releases intracellular silica that is further taken up by other AMs [7,8]; (2) Silicic acid produced by dissolved silica destroys the stability of the AM lysosomal membrane. Hydrolase released by the disrupted lysosome penetrates the cytoplasm overly and ultimately leads to AM death [9,10,11]; (3) Dead AMs can release a series of inflammatory factors, causing pulmonary inflammatory damage [12]. Correspondingly, AMs gather at the injured pulmonary tissue and stimulate fibroblasts to transform into myofibroblasts, leading to excessive deposition of the extracellular matrix and eventual silicosis fibrosis [13,14,15]. These steps are not necessarily executed in order or parallel strictly, and they are interspersed and connected to cause silicotic fibrosis.

### 1.2. Programmed Death of Various Types of Cells Has Different Significance for the Occurrence and Development of Silicosis

As mentioned above, transforming growth factor-β (TGF-β) secretion stimulates fibroblasts to transform into myofibroblasts for collagen synthesis, extracellular matrix deposition, and eventual silicosis fibrosis formation [16]. The inhibited activity of autophagy has been observed in TGF-β-treated fibroblasts. *MiR-449a* induced autophagy activity and reduced Bcl-2 level in silica-activated fibroblasts or a silicosis mice model [17]. Meanwhile, m*iR-326* also promotes autophagy activity by targeting polypyrimidine tract-binding protein 1 (PTBP1) [18]. Their over-expression alleviated both the distribution and severity of lung lesions. HECT domain-containing protein 1 (HECTD1) is an E3 ubiquitin-protein, which has been proven to be involved in functional cellular changes in silicosis. Either *circHECTD1* over-expression or *HECTD1* knockdown inhibited silica-induced fibroblast activation, proliferation, and migration via regulating the autophagy activity of fibroblasts [19]. In summary, related research concerning non-coding RNA with its targeted protein, which can regulate autophagy activity, may shed new light on the therapeutic methods of silicosis. Moreover, Rho GDP-dissociation inhibitor α (RhoGDIα) knockdown inhibited collagen deposition through promoting apoptosis of myofibroblasts [20]. Overall, unlike AM, related research based on silica-activated fibroblasts or myofibroblasts should promote autophagy or apoptosis to seek promising intervention methods of silicosis. Notably, in this review, we attached much attention to related studies based on AMs and lung epithelial cells (detailed below). They are both critical targeted cells.

## 2. Autophagy Is an Essential Way of Programmed Cell Death during Silicotic Progression

**Figure 1:**
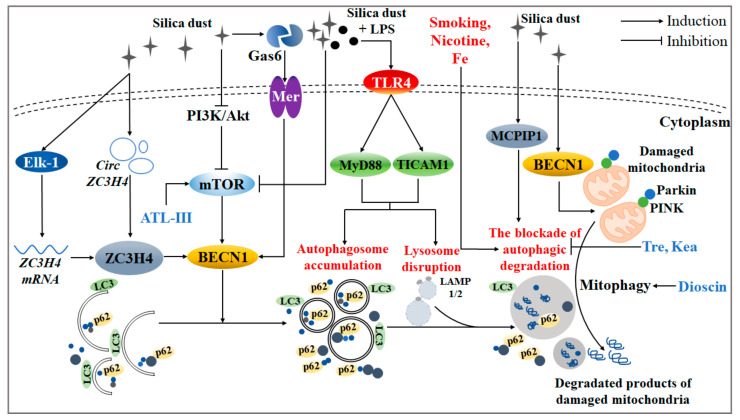
Related molecular mechanisms and effects during the process of silica-induced autophagy. Silica invasion led to the accumulation of autophagosomes and the disruption of lysosomes, thereby breaking the function of autophagic degradation through the PI3K/Akt/mTOR signaling pathway. Under silica circumstances, ZC3H4 and MCPIP1 both activated autophagy activity. Concretely, ZC3H4 was enhanced not only by the up-regulation of *circZC3H4*, but also by the transcription of *ZC3H4 mRNA* via phosphorylation of Elk-1. Furthermore, the interaction of Gas6 and its receptor, Mer, boosted autophagy activity. In addition, Fe atoms were found to be accumulated on the surface of silica, which further magnified the pathological damage of the autophagic process. LPS, nicotine, and habitual smoking all led to the blockade of autophagic degradation. Specifically, TLR4/Myd88 or TLR4/TICAM signaling pathways might be involved in the autophagy induced by silica together with LPS. Particularly, natural products like tre and kea had a protective function in the degradation process of autophagic substrates. When silica invaded, dioscin removed redundant damaged mitochondria through AM mitophagy, and ATL-III protected AM autophagic degradation via an mTOR-dependent signaling pathway.

Autophagic cell death (i.e., autophagy) is named type II programmed cell death. It is a biological phenomenon that exists widely in eukaryotic cells, which has a powerful ability to “digest” the “waste” of cells [21,22]. Existing studies have shown that the mechanism of silica-induced autophagy is quite complex, involving a series of autophagy-related signaling pathways and regulatory proteins (Figure 1). That understanding of how these signals are linked to silica-induced autophagy may further improve treatment therapies for silicosis.

### 2.1. Autophagy Plays a Complex Role during the Development of Silicosis via Involvement in Multiple Signaling Pathways

Silica has been proven to regulate autophagy activity via the phosphatidylinositol 3 kinase (PI3K)/protein kinase B (PKB/Akt)/mammalian target of rapamycin (mTOR) signaling pathway. Recent studies have shown that, through the utilization of mTOR inhibitor rapamycin (Rapa), autophagy alleviates silica-induced AM apoptosis [23]. Meanwhile, autophagy reduces the expression of tumor necrosis factor-α (TNF-α) and TGF-β in AMs treated with silica [24]. On the one hand, these findings suggest that the activation of AM autophagy can protect against the silica-induced excessive cell apoptosis or inflammatory response. On the other hand, an mTOR signaling pathway may be a critical point for the mechanism of autophagy. Especially, an active ingredient of the natural plant Atractylodes macrocephala Koidz, Atractylenolide III (ATL-III), accelerated the process of autophagic degradation via fostering the mTOR-dependent signaling pathway [25]. ATL-III may be the potential natural mTOR activator that has been discovered. Therefore, the development of natural or synthetic drugs targeting mTOR may be a promising method for silicosis treatment in clinical settings.

As described, the basic autophagy level has a compensatory protective function for silica invasion; however, cells seem to have the ability to sense the stress caused by silica, which further leads to dysregulation of related signaling pathways and even cell death via the abnormal occurrence of autophagy. Accumulated autophagosomes and damaged lysosomes in the AM of silicosis patients have been observed previously [26], implying that silica disrupts the normal process of AM autophagic degradation. This may be an indispensable feature of abnormal autophagy caused by excessive silica invasion. Moreover, mitophagy also participates in the mechanism of the silica-induced pulmonary toxic effect. When invading the alveoli, silica-activated AM produces mitochondria ROS (mtROS), reduces ATP contents, and breaks mitochondria function. In response to such pathological pulmonary damage, the expression of PINK and Parkin is decreased, which are regulated by BECN1. Meanwhile, the deficiency of BECN1, targeted by *microRNA-1224-5p*, triggered mitophagy disruption under silica circumstances [27]. Furthermore, dioscin, the main ingredient of Dioscoreaceae, eliminated damaged mitochondria via protecting impaired mitophagy against silica attack [23,28].

Gram-negative bacteria exist in the Chinese mine working environment widely. Correspondingly, lipopolysaccharide (LPS) has been detected in the bronchoalveolar lavage fluid of silicosis patients. LPS further intensifies the blockade of AM autophagic degradation of silicosis patients via the activation of toll-like receptor 4 (TLR4)/myeloid differentiation factor 88 (MyD88) and the toll-like receptor adaptor molecule 1 (TLRAM1) signaling pathway or inhibition of mTOR signaling pathway [26]. Surprisingly, extensive research has demonstrated that the lung of a healthy body is in a microbial flora balance instead of being “aseptic” [29,30]. In the future, more importance should be attached to the pulmonary toxic effect induced by silica combined with LPS or other classical bacterial flora. It can be speculated that LPS, the vital component of Gram-negative bacteria, may reflect the complex components of the actual working environment to some extent.

### 2.2. Several Proteins Have Been Proven to Participate in the Regulation of Silica-Induced Autophagy

Accumulating evidence has shown that some CCCH zinc finger protein family members have been discovered to have a potential regulatory function for autophagy induced by silica. For instance, after silica treatment, monocyte chemoattractant protein-1-induced protein 1 (MCPIP1) activated macrophage autophagy activity, further aggravating the silicosis progression through the p53 signaling pathway. Meanwhile, *MCPIP1-siRNA* reversed silica-induced autophagy and apoptosis of macrophages [31,32]. Like MCPIP1, zinc finger CCCH-type containing 4 protein (ZC3H4) greatly encouraged the autophagy level, further affecting epithelial-mesenchymal transition (EMT) in endothelial cells [33,34,35]. Concretely, ZC3H4 can be activated in multiple ways: (1) the competitive combination of *circZC3H4* and *miR-212* [36]; (2) the transcription of *ZC3H4 mRNA* (which may be caused by phosphorylation of Ets-like protein-1 (Elk-1)) [37]. Taken together, ZC3H4 with its related upstream regulators may be useful as a novel autophagy regulatory target for silicosis intervention. Furthermore, an increased secretion of growth arrest-specific protein 6 (Gas6) and its typical receptor Mer has been observed in the bronchoalveolar lavage fluid of the silicosis mice model [38]. Furthermore, the destruction of autophagic degradation was ameliorated in *Gas6*^−/−^ or *Mer*^−/−^ mice exposed to silica [39]. Overall, these discovered proteins involved in the autophagy regulation may provide a new idea for treating silicosis patients.

### 2.3. The Complex Components of the Actual Environment Exposed to Silica Dust Is Still Being Explored

In addition to LPS, there has been an increasing interest in other “stimuli” in the workplace under silica circumstances. Compared to those with a low smoking index or no smoking, the AMs of silicosis patients with a high smoking index had a greater level of autophagic degradation disruption [40]. Furthermore, nicotine, the main ingredient of smoking, accelerated the disrupted autophagic degradation, leading to AM apoptosis finally [41]. Therefore, the prohibition of tobacco smoke or secondhand smoke may also be necessary as a “safeguard” for workers exposed to silica [42].

Intriguingly, Fe atoms were found to be accumulated on the surface of silica. Their size and number were increased with the aggravation of pathological changes of the silicosis rat model. Meanwhile, sequestosome1 (SQSTM1/p62) was accumulated around the silica while not expressed in control mice [43]. Thus, the normal silicosis animal model constructed by single-crystalline SiO_2_ may not be appropriate. More attention should be paid to the combined pulmonary toxicity by SiO_2_ and its surface adsorbent. The relationship between these exogenous stimuli and autophagy in the pathological development of silicotic fibrosis should be examined deeply.

### 2.4. The Process of Autophagic Degradation, Not Autophagy Activity, Should Be Given More Focus during the Process of Silica-Induced Autophagy

Accumulating studies seem to suggest that the change of autophagy activity fails to explain the role that autophagy plays in silica-induced pulmonary fibrosis. For instance, dioscin might delay the progression of silicosis via the activation of autophagy to eliminate damaged mitochondria [23,28]. However, enhanced autophagy activity aggravated silica-induced macrophage apoptosis in the MCPIP1 deficiency of mice. Herein, we support a hypothesis: the degree of autophagic degradation, not the change of autophagy activity, may better reflect the autophagy regulatory mechanism of certain endogenous or exogenous substances in silicotic fibrosis. Correspondingly, many natural products have attracted much attention in studies of pulmonary fibrosis [44]. They may also be protective anti-fibrotic components of silicosis by targeting autophagic degradation, such as ATL-III, dioscin, trehalose (tre, a non-reducing disaccharide), and kaempferol (kae, a flavonoid that exists in many plants and fruits) [23,45,46]. In the future, the molecular mechanism of some natural products with autophagic regulation should be taken as the starting point for exploring the interventions for silicosis.

## 3. Apoptosis and Pyroptosis Are Both Associated with Toxic Effects Induced by Silica

**Figure 2:**
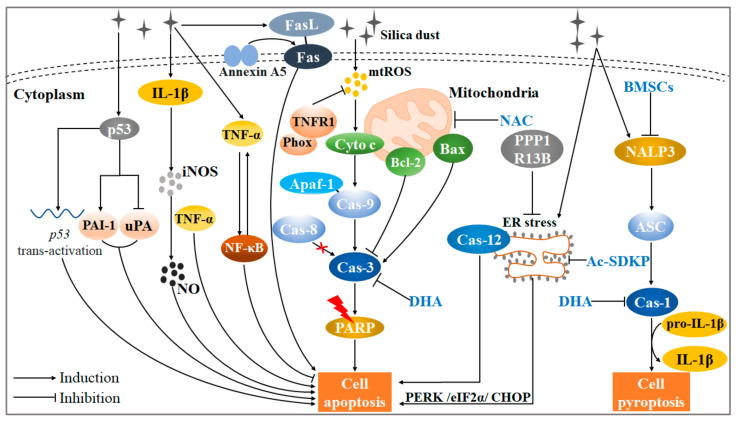
A complex regulatory network of initiation of silica-induced apoptosis or pyroptosis. Normally, when silica invades the alveoli, mtROS is produced and augments the formation of the cas-9-Apaf-1 complex. Furthermore, cas-3 is activated to crack PARP, leading to cell apoptosis finally. In such a mitochondrial-dependent apoptotic pathway, the increased ratio of Bax/Bcl-2 aggravates the cas-3 activation. Moreover, the Fas/FasL signaling pathway, induced by upstream factor Annexin A5, also mediates silica-induced apoptosis. p53 induces apoptosis, not via the inhibition of uPA and induction of PAI-1, but via p53 trans-activation. Another member of the p53 family, PPP1R13B, promotes over-activation of ER stress via the PERK/eIF2α/CHOP signaling pathway, further initiating silica-induced apoptosis. Similarly, it also can be activated by ER stress-related protein cas-12. Silica-induced apoptosis is still induced by the IL-1β-iNOS-NO cascade reaction. Notably, TNF-α activation aggravates cell apoptosis. However, TNF-α also stimulates the NF-κB signaling pathway to alleviate cell apoptosis against silica invasion. The bidirectional role of TNF-α deserves further discussion. It is worth noting that NAC and DHA have potential anti-apoptotic effects activated by silica. In addition to apoptosis, silica can stimulate the interaction of NALP3 and ASC, and subsequent NALP3-ASC complex activates cas-1. The cas-1 activation leads to the transformation from pro-IL-1β to IL-1β, further executing cell pyroptosis. BMSCs might mitigate cell pyroptosis via inhibition of NALP3.

The apoptosis occurring at an early stage of the silicosis progression has a compensatory function to clear up injured cells and inflammation, assisting in the lung tissue remodeling. However, when AM has a failed phagocytosis or abnormalities in the clearance of the apoptotic AMs, the level of AM apoptosis will escalate gradually, aggravating silicotic fibrosis eventually [47]. Additionally, pyroptosis is a way of host cell death recently discovered, whose stimulating factors include microbial infection, damage-associated molecular pattern, products of ischemic necrosis, etc. Typically, LRR and PYD domains-containing protein 3 (NALP3) combines with apoptosis-associated speck-like protein containing a CARD (ASC) through PYD domains. The NALP3-ASC complex propels caspase-1 to execute pyroptosis. Particularly, pyroptosis is essentially cell inflammatory necrosis mediated by gasdermin D (GSDMD) [48,49,50]. Recent evidence suggests that silica may also be a “red flag” for stimulating cell pyroptosis on a pathological level [51]. This review summarizes the current molecular mechanism of silica-induced apoptosis or pyroptosis (Figure 2).

### 3.1. Silica-Induced Cell Apoptosis Can Be Mediated by Intrinsic or Exogenous Signaling Pathways

Normally, silica boosts mitochondria to produce mtROS and releases cytochrome c (cyto-c) [52,53]. Cyto-c binds apoptotic protease activating factor-1 (Apaf-1) to initiate a caspase cascade reaction: the cyto-c/Apaf-1 complex activates caspase-9 (not caspase-8) then caspase-3, and the latter can crack poly ADP-ribose polymerase (PARP), further leading to DNA fragmentation (a characteristic of cell apoptosis) [47]. In addition, the interaction between TNF receptor 1 (TNFR1) and NADPH oxidase (Phox) may reduce the mtROS production, alleviating macrophage apoptosis [54]. Our previous study had found that the decreased ratio of Bcl-2/Bax resulted in the caspase-3 activation in the silicosis mice model [55,56]. Moreover, mitochondria-mediated apoptosis occurred in mouse macrophage line MH-S cells with silica exposure, which manifested as the appearance of subdiploid cell fragments, accompanied by the activation of caspase-3 and caspase-9 [57]. The caspase-3 expression was also enhanced in LPS-intervened AMs of silicosis patients or silicosis mice lung tissue [26,58], suggesting that caspase-3 might be a critical center factor during cell apoptosis progress in silicosis. Notably, N-acetylcysteine (NAC) might alleviate the progression of silicosis via regulating the mitochondria-mediated apoptotic pathway [59].

In addition to the intrinsic apoptotic signaling pathway, the Fas/FasL signaling pathway is also one of the major pathways that regulates apoptosis [60]. Fas and the related death-ligand FasL combination can trigger the polymerization of Fas monomers into Fas trimer complexes, and the latter executes cell apoptosis through its death domain [61]. Fas and TNF-α expression in AMs from silicosis patients were significantly higher than those from healthy volunteers or observed objects [62,63]. Another study showed that comparing normal silicotic mice, the FasL^−/−^ silicotic mice did not exhibit obvious inflammatory injury. Furthermore, treatment with anti-FasL Antibodies inhibited the release of TNF-α in the AMs of a silicosis mice model [64]. Simultaneously, annexin A5 may be an upstream protein of Fas/FasL apoptotic pathways, promoting macrophage activation in silica-induced lung fibrosis [65]. In brief, the intrinsic or exogenous pathways both play a crucial role in silica-induced cell apoptosis. Future research should focus on the related molecular mechanism.

### 3.2. p53 and Its Family Member May Be a Critical Regulator in the Silica-Induced Cell Apoptotic Process

Many investigators have demonstrated that p53 may participate in silica-induced cell apoptosis. Specifically, silica promoted p53 trans-activation through ser-392 p53 phosphorylation and p53 accumulation in embryo fibroblasts [66,67]. Interestingly, the relationship among plasminogen activator inhibitor-1 (PAI-1), urokinase-type plasminogen activator (uPA), and p53 in pulmonary fibrosis has attracted much attention [68,69]. Silica-induced p53 accumulation not only activated PAI-1 expression, but also inhibited uPA expression, inducing apoptosis of lung epithelial cells. Correspondingly, the deficiency of p53 or PAI-1 alleviated cell apoptosis in silica-exposed mice [70]. However, there is no interference among p53 trans-activation, uPA mRNA, and PAI-1 mRNA [71]. These results above suggest that inhibition of p53 expression alone or disruption of p53-fibrinolytic system crosstalk may serve as a novel intervention strategy to prevent silicosis.

PPP1R13B, a major member of the apoptosis-stimulating proteins of the p53 family, may perform an anti-apoptosis function through alleviating endoplasmic reticulum (ER) stress [72,73]. Moreover, a study has shown that continuous silica invasion leads to A549 cell apoptosis induced by excessive ER stress, reflected in the phosphorylation of protein kinase RNA-like endoplasmic reticulum kinase (PERK), eukaryotic initiation factor α (eIF2α), and the up-regulation of CHOP and Caspase-12. Intriguingly, N-acetyl-seryl-aspartyl-lysyl-proline (Ac-SDKP), a physiological regulatory peptide factor, may alleviate A549 cell apoptosis via the PERK/eIF2α/CHOP signaling pathway [74].

### 3.3. Other Cytokines Form a Complex Apoptotic Network in Silicosis

Currently, the pathological effects of the nuclear factor kappa-B (NF-κB) and TNF-α in silica-induced apoptosis remain controversial. TNF-α has been recognized as a biomarker for the early diagnosis of silicosis [75]. The enhanced TNF-α production was observed in macrophages in response to silica activation, fostering macrophage apoptosis. Furthermore, anti-TNF-α antibodies or soluble TNF receptors improved pulmonary fibrosis in silica-exposed mice [76,77,78]. Silica was able to induce TNF-α transcription via the NF-κB activation. TNF-α also stimulated the NF-κB signaling pathway to protect the cell apoptosis against silica invasion in RAW 264.7 murine macrophages. Such a mechanism may be compensatory protection for lung tissue damage. However, excessive cell apoptosis and pulmonary inflammatory response occur with the over-activation of NF-κB [79,80]. Therefore, antagonism of TNF-α may not constitute an appropriate clinical target in silicosis. The balance between NF-κB activity and TNF-α expression may decide the degree of cell apoptosis and cell fate. Future research should consider the bidirectional role of TNF-α in silica-induced apoptosis more carefully.

It has been recognized that silica-activated AMs release many cytokines, like TNF-α, interleukin-1β (IL-1β), and IL-6, etc. [55], which is the basis of silicosis fibrosis. Among them, IL-1β not only induces an inflammatory response, but regulates macrophage apoptosis [8]. Specifically, IL-1β activated inducible nitric oxide synthase (iNOS), thereby promoting nitric oxide (NO) production, which was also a critical substance to induce apoptosis [81]. Notably, the Fas/FasL can release IL-1β and TNF-α [82,83], implying that the crosstalk between the apoptotic mechanism and cytokine release remains to be elucidated in more detail.

### 3.4. Silica Is Also an “Irritant” for the Execution of Cell Pyroptosis

NLRP3 and its downstream factors, caspase-1, IL-1β, and IL-18, were greatly expressed in the lung tissue of rats with silicosis [84], while the inhibition or deficiency of NLRP3 alleviated EMT in lung epithelial cells or inflammation in macrophages [85,86]. These findings confirm the view that silica may promote caspase-1 to selectively cleave pro-IL-1β to mature IL-1β, thereby inducing cell death via the pyroptotic pathway [87,88]. Furthermore, bone marrow mesenchymal stem/stromal cells (BMSCs) transplantation may have a potential anti-fibrotic effect for silicosis [89,90,91]. BMSC treatment attenuated NLRP3 and caspase-1 expression, relieving lung inflammatory infiltrates and collagen deposition effectively in the silicosis rat model. Additionally, the levels of BECN1, microtubule-associated protein 1 light chain 3 (LC3), and p62 did not change obviously in this study, implying that autophagy is not associated with the potential anti-silicotic effect of BMSC transplantation [92]. On the contrary, another study indicated that LC3 and BECN1 were decreased in the silicosis rat model with BMSCs. It indicates that the mitigation of pulmonary tissue damage may be caused by the inhibition of autophagy activity [93]. Such an opposite conclusion may be due to the difference in the administration time and dosage of BMSCs. The significance of autophagy, pyroptosis, and BMSCs in the progression of silicosis is still an issue for future research to explore.

However, contrary to the view above, some researchers have supported a proposal that silica alone did not activate NRLP3 inflammasome-directed pyroptosis, because IL-1β release did not change dramatically, although caspase-1 is activated in AM with a single SiO_2._ Meanwhile, NLRP3 activation, subsequent ASC oligomerization, and caspase-1 activation were observed in AM with LPS priors to silica treatment. The reason may be that NLRP3 expression requires priming with microbial ligands such as LPS or endogenous cytokines, not inducing IL-1β release in unprimed macrophages. Furthermore, docosahexaenoic acid (DHA) inhibited cell pyroptosis in silica-activated AMs treated with LPS [94,95]. In summary, whether single or combined silica can induce pyroptosis in silicosis fibrosis still needs to be further explored. The significance behind this may reflect that the components of silica in an actual working environment are much more complicated than imagined.

## 4. The Crosstalk among Autophagy, Apoptosis, and Pyroptosis in Silicotic Fibrosis Needs to Be Further Discussed

As described before, autophagy can act as an enabler or antagonist of apoptosis to impede or promote cell survival [96]. Moreover, there is the crosstalk that exists between autophagy and apoptosis. Bcl-2 is a critical anti-apoptotic protein in silica-induced autophagy. The formation of the BECN1-PIK3C3 complex is quite essential for the extension of the autophagosome bilayer membrane, while Bcl-2 binds to BECN1 then inhibits the interaction of BECN1 and PI3KC3 or the phosphorylation of PI3KC3 [97]. A study has demonstrated that silica induces the BECN1–PIK3C3 complex but reduces the BECN1–Bcl-2 complex, further escalating autophagy level [98]. Moreover, Bcl-2-binding component 3 (BBC3), a potent activator of apoptosis that also belongs to the Bcl-2 family [99], may inhibit the binding of BECN1 and Bcl-2 competitively, thereby facilitating silica-induced macrophage autophagy activity [100]. In addition, a widely accepted stimulating factor to mediate apoptosis, ER stress, may also stimulate autophagy activity in the development of silicosis, caused by the excessive accumulation of unfolded proteins in the endoplasmic reticulum [33,101].

**Figure 3:**
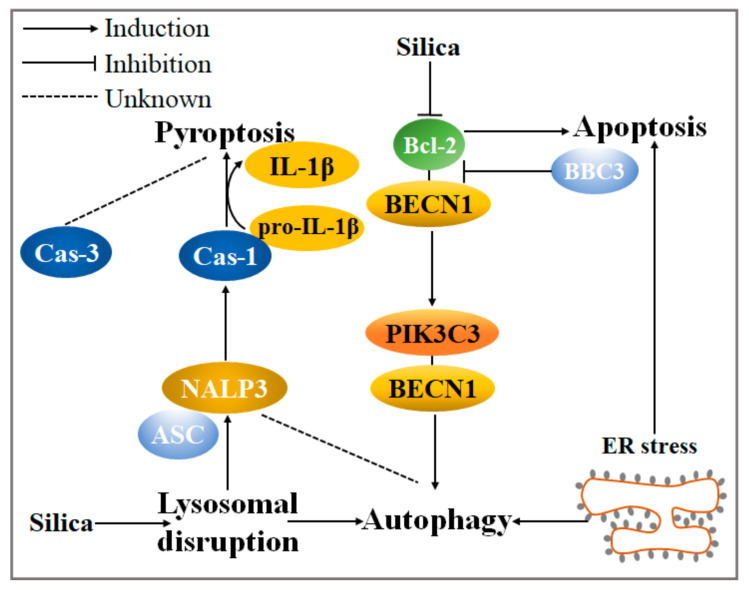
The current molecular mechanism of crosstalk among autophagy, apoptosis, and pyroptosis under silica circumstances. Two important members of the Bcl-2 apoptotic protein family, Bcl-2 and BBC3, activate autophagy via reducing the Bcl-2–BECN1 complex in silica-induced pulmonary fibrosis. Autophagy activity is also stimulated by silica-induced ER stress. Lysosomal disruption, a critical characteristic of silica-induced autophagy, could foster the formation of the NALP3–ASC complex. It further activates cas-1, boosting the transformation from pro-IL-1β to IL-1β and executing pyroptosis. Notably, the relationship between autophagy and NALP3 or the mechanism of cas-3-mediated pyroptosis deserves more exploration.

Admittedly, there is also an interaction between autophagy and pyroptosis (Figure 3). NALP3, IL-1β, and caspase-1 were activated via lysosomal disruption caused by macrophage phagocytosis for silica [102]. Furthermore, the deficiency of autophagy by *Atg5* knockout activated NLRP3 inflammasome activity [103]. These findings indicate that pyroptosis may be closely associated with autophagy. Meanwhile, like apoptosis, pyroptosis is also a critical pathological process in silicosis. In the future, silica-induced pyroptosis can be regarded as an indicator together with apoptosis in the studies of the autophagy-related mechanism to reflect the silicosis progression. In addition to apoptosis, it is further possible that caspase-3 can cleave the GSMDE N-terminal fragment, which forms pores on the plasma membrane to mediate pyroptosis [104,105]. Therefore, it will be desirable in future studies to investigate the relationship between caspase-3 and pyroptosis during the development of silicosis.

## 5. Conclusions

Silicosis has been a severe occupational hazard disease until now. The proposed hypothesis cannot explain the pathological mechanism of silicosis perfectly, even though it has been gradually elucidated. From the perspective of programmed cell death, we summarized an amount of autophagic or apoptotic signaling pathways or regulatory proteins in silicosis pathogenesis. Moreover, as a “dangerous flag”, silica can also exacerbate pyroptosis. Herein, we proposed some views: (1) The blockade of autophagic degradation is characteristic of abnormal autophagy caused by silica. The degree of the blockade of autophagic degradation, not autophagy activity, may reflect positive or negative effects of autophagy on silicosis better when discussing the function of the exogenous toxic substance or potential protective agent on silica-induced autophagy; (2) Silicotic progression depends on the combined effect of silica and exogenous irritants, not silica alone. Furthermore, the silicosis animal or cell model should be established more cautiously due to the complex composition of silica; (3) Many related mechanisms, like the bidirectional effect of TNF-α in silica-induced apoptosis, or the regulatory function by caspase-3 on pyroptosis deserves further discussion; (4) Pyroptosis may be a potential mechanism during the development of silicosis, and it should also be regarded as an “outcome variable” to assess the severity of pathological changes in silicosis. As also recommended above, these opinions above may provide some promising enlightenment for future interventions or treatment for silicosis.

## Data Availability

Not applicable.

## Abbreviations

Ac-SDKP	N-acetyl-seryl-aspartyl-lysyl-proline
AM	Alveolar macrophage
Apaf-1	Apoptotic protease activating factor-1
ASC	Apoptosis-associated speck-like protein containing a CARD
ATL-III	Atractylenolide III
BBC3	Bcl-2-binding component 3 (BBC3)
BMSCs	Bone marrow mesenchymal stem/stromal cells (BMSCs)
Cyto-c	Cytochrome c
DHA	Docosahexaenoic acid
eIF2α	eukaryotic initiation factor α
Elk-1	Ets-like protein-1 (Elk-1)
EMT	Epithelial-mesenchymal transition
ER	Endoplasmic reticulum
Gas6	Growth arrest-specific protein 6
GSDMD	Gasdermin D
HECTD1	HECT domain-containing protein 1
IL-1β	Interleukin-1β
iNOS	Inducible nitric oxide synthase
Kea	Kaempferol
LC3	Microtubule-associated protein 1 light chain 3
LPS	Lipopolysaccharide
MCPIP1	Monocyte chemoattractant protein-1-induced protein 1
mTOR	mammalian target of rapamycin
mtROS	mitochondria ROS
MyD88	Myeloid differentiation factor 88
NAC	N-acetylcysteine
NALP3	LRR and PYD domains-containing protein 3
NF-κB	Nuclear factor kappa-B
NO	Nitric oxide
PAI-1	Plasminogen activator inhibitor-1
PARP	Poly ADP-ribose polymerase
PCD	Programmed cell death
PERK	Protein kinase RNA-like endoplasmic reticulum kinase
Phox	NADPH oxidase
PI3K	Phosphatidylinositol 3 kinase
PKB/Akt	Protein kinase B
PTBP1	Polypyrimidine tract-binding protein 1
Rapa	Rapamycin
RhoGDIα	Rho GDP-dissociation inhibitor α
SQSTM1/p62	Sequestosome1
TGF-β	Transforming growth factor-β
TLR4	Toll-like receptor 4
TLRAM1	Toll-like receptor adaptor molecule 1
TNF-α	Tumor necrosis factor-α
TNFR1	TNF receptor 1
Tre	Trehalose
uPA	urokinase-type plasminogen activator
ZC3H4	Zinc finger CCCH-type containing 4 protein

## References

[1] Gaida A., Piasco D. (1963). The diffuse interstitial fibrosis component of pulmonary silicosis. Minerva Med..

[2] Barnes H., Goh N.S.L., Leong T.L., Hoy R. (2019). Silica-associated lung disease: An old-world exposure in modern industries. Respirology.

[3] Galluzzi L., Vitale I., Aaronson S.A., Abrams J.M., Adam D., Agostinis P., Alnemri E.S., Altucci L., Amelio I., Andrews D.W. (2018). Molecular mechanisms of cell death: Recommendations of the Nomenclature Committee on Cell Death 2018. Cell Death Differ..

[4] Brumatti G., Salmanidis M., Ekert P.G. (2010). Crossing paths: Interactions between the cell death machinery and growth factor survival signals. Cell Mol. Life Sci..

[5] Tan S., Chen S. (2021). Macrophage autophagy and silicosis: Current perspective and latest insights. Int. J. Mol. Sci..

[6] Lee S., Honda M., Yamamoto S., Kumagai-Takei N., Yoshitome K., Nishimura Y., Sada N., Kon S., Otsuki T. (2019). Role of nephronectin in pathophysiology of silicosis. Int. J. Mol. Sci..

[7] Joshi G.N., Knecht D.A. (2013). Silica phagocytosis causes apoptosis and necrosis by different temporal and molecular pathways in alveolar macrophages. Apoptosis.

[8] Rimal B., Greenberg A.K., Rom W.N. (2005). Basic pathogenetic mechanisms in silicosis: Current understanding. Curr. Opin. Pulm. Med..

[9] Carlisle E.M. (1975). Silicon. Nutr. Rev..

[10] Wang L.Y., Scabilloni J.F., Antonini J.M., Rojanasakul Y., Castranova V., Mercer R.R. (2006). Induction of secondary apoptosis, inflammation, and lung fibrosis after intratracheal instillation of apoptotic cells in rats. Am. J. Physiol. Lung C.

[11] Hamilton R.F., Thakur S.A., Holian A. (2008). Silica binding and toxicity in alveolar macrophages. Free Radic. Biol. Med..

[12] McGrath K.C., Li X.H., McRobb L.S., Heather A.K., Gangoda S.V.S. (2015). Inhibitory effect of a French maritime pine bark extract-based nutritional supplement on TNF-α-induced inflammation and oxidative stress in human coronary artery endothelial cells. Evid. Based Complement. Altern. Med..

[13] Kehlet S.N., Willumsen N., Armbrecht G., Dietzel R., Brix S., Henriksen K., Karsdal M.A. (2018). Age-related collagen turnover of the interstitial matrix and basement membrane: Implications of age- and sex-dependent remodeling of the extracellular matrix. PLoS ONE.

[14] Quan B., Zhang H., Xue R. (2019). miR-141 alleviates LPS-induced inflammation injury in WI-38 fibroblasts by up-regulation of NOX2. Life Sci..

[15] Lurton J., Soto H., Narayanan A.S., Raghu G. (1999). Regulation of human lung fibroblast C1q-receptors by transforming growth factor-beta and tumor necrosis factor-alpha. Exp. Lung Res..

[16] Arcangeli G., Cupelli V., Giuliano G. (2001). Effects of silica on human lung fibroblast in culture. Sci. Total Environ..

[17] Han R., Ji X., Rong R., Li Y., Yao W., Yuan J., Wu Q., Yang J., Yan W., Han L. (2016). MiR-449a regulates autophagy to inhibit silica-induced pulmonary fibrosis through targeting Bcl2. J. Mol. Med..

[18] Xu T., Yan W., Wu Q., Xu Q., Yuan J., Li Y., Li P., Pan H., Ni C. (2019). MiR-326 Inhibits Inflammation and Promotes Autophagy in Silica-Induced Pulmonary Fibrosis through Targeting TNFSF14 and PTBP1. Chem. Res. Toxicol..

[19] Chu H., Wang W., Luo W., Zhang W., Cheng Y., Huang J., Wang J., Dai X., Fang S., Chao J. (2019). CircHECTD1 mediates pulmonary fibroblast activation via HECTD1. Ther. Adv. Chronic Dis..

[20] Wei Z., Xu H., Zhang Y., Yi X., Yang X., Chen Y., Mao N., Li S., Xu D., Li S. (2019). Rho GDP dissociation inhibitor α silencing attenuates silicosis by inhibiting RhoA/Rho kinase signalling. Exp. Cell Res..

[21] Hotchkiss R.S., Strasser A., McDunn J.E., Swanson P.E. (2009). Cell death. N. Engl. J. Med..

[22] Glick D., Barth S., Macleod K.F. (2010). Autophagy: Cellular and molecular mechanisms. J. Pathol..

[23] Du S., Li C., Lu Y., Lei X., Zhang Y., Li S., Liu F., Chen Y., Weng D., Chen J. (2019). Dioscin alleviates crystalline silica-induced pulmonary inflammation and fibrosis through promoting alveolar macrophage autophagy. Theranostics.

[24] Li N., Shi F., Wang X., Yang P., Sun K., Zhang L., Hao X., Li X., Li J., Li Y. (2021). Silica dust exposure induces pulmonary fibrosis through autophagy signaling. Environ. Toxicol..

[25] Chen S., Tang K., Hu P., Tan S., Yang S., Yang C., Chen G., Luo Y., Zou H. (2021). Atractylenolide III alleviates the apoptosis through inhibition of autophagy by the mTOR-dependent pathway in alveolar macrophages of human silicosis. Mol. Cell. Biochem..

[26] Chen S., Yuan J., Yao S., Jin Y., Chen G., Tian W., Xi J., Xu Z., Weng D., Chen J. (2015). Lipopolysaccharides may aggravate apoptosis through accumulation of autophagosomes in alveolar macrophages of human silicosis. Autophagy.

[27] Wu Q., Xu T., Liu Y., Li Y., Yuan J., Yao W., Xu Q., Yan W., Ni C. (2017). miR-1224-5p mediates mitochondrial damage to affect silica-induced pulmonary fibrosis by targeting BECN1. Int. J. Mol. Sci..

[28] Li C., Lu Y., Du S., Li S., Zhang Y., Liu F., Chen Y., Weng D., Chen J. (2017). Dioscin exerts protective effects against Crystalline silica-induced pulmonary fibrosis in mice. Theranostics.

[29] Dickson R.P., Erb-Downward J.R., Martinez F.J., Huffnagle G.B. (2016). The microbiome and the respiratory tract. Annu. Rev. Physiol..

[30] Charlson E.S., Bittinger K., Chen J., Diamond J.M., Li H., Collman R.G., Bushman F.D. (2012). Assessing bacterial populations in the lung by replicate analysis of samples from the upper and lower respiratory tracts. PLoS ONE.

[31] Liu H., Fang S., Wang W., Cheng Y., Zhang Y., Liao H., Yao H., Chao J. (2016). Macrophage-derived MCPIP1 mediates silica-induced pulmonary fibrosis via autophagy. Part. Fibre Toxicol..

[32] Wang X., Zhang Y., Zhang W., Liu H., Zhou Z., Dai X., Cheng Y., Fang S., Zhang Y., Yao H. (2016). MCPIP1 regulates alveolar macrophage apoptosis and pulmonary fibroblast activation after in vitro exposure to silica. Toxicol. Sci..

[33] Jiang R., Han L., Gao Q., Chao J. (2021). ZC3H4 mediates silica-induced EndoMT via ER stress and autophagy. Environ. Toxicol. Pharmacol..

[34] Jiang R., Zhou Z., Liao Y., Yang F., Cheng Y., Huang J., Wang J., Chen H., Zhu T., Chao J. (2019). The emerging roles of a novel CCCH-type zinc finger protein, ZC3H4, in silica-induced epithelial to mesenchymal transition. Toxicol. Lett..

[35] Yi J.H., Zhang Z.C., Zhang M.B., He X., Lin H.R., Huang H.W., Dai H.B., Huang Y.W. (2021). Role of epithelial-to-mesenchymal transition in the pulmonary fibrosis induced by paraquat in rats. World J. Emerg. Med..

[36] Jiang R., Gao Q., Chen M., Yu T. (2020). Elk-1 transcriptionally regulates ZC3H4 expression to promote silica-induced epithelial-mesenchymal transition. Lab. Investig..

[37] Yang X., Wang J., Zhou Z., Jiang R., Huang J., Chen L., Cao Z., Chu H., Han B., Cheng Y. (2018). Silica-induced initiation of circular ZC3H4 RNA/ZC3H4 pathway promotes the pulmonary macrophage activation. FASEB J..

[38] Li W., Xie L., Ma J., Yang M., Wang B., Xu Y., Fan L., Mu G., Shi T., Chen W. (2019). Genetic loss of Gas6/Mer pathway attenuates silica-induced lung inflammation and fibrosis in mice. Toxicol. Lett..

[39] Li W., Xie L., Ma J., Cheng M., Fan L., Xu Y., Wang B., Chen W. (2021). Gas6 or Mer deficiency ameliorates silica-induced autophagosomes accumulation in mice lung. Toxicol. Lett..

[40] Chen M.K., Tan S.Y., Wang Y.R., Li S.H., Chen G., Chen S. (2020). The study of smoking impact on autophagy in alveolar macrophages of human silicosis. Zhonghua Lao Dong Wei Sheng Zhi Ye Bing Za Zhi.

[41] Chen S., Tan S., Yang S., Chen G., Zhu L., Sun Z., Li H., Yao S. (2020). Nicotine induces apoptosis through exacerbation of blocked alveolar macrophage autophagic degradation in silicosis. Toxicol. Lett..

[42] Sager T.M., Umbright C.M., Mustafa G.M., Yanamala N., Leonard H.D., McKinney W.G., Kashon M.L., Joseph P., McKinney W.G. (2020). Tobacco smoke exposure exacerbated crystalline silica-induced lung toxicity in rats. Toxicol. Sci..

[43] Shimizu Y., Dobashi K., Nagase H., Ohta K., Sano T., Matsuzaki S., Ishii Y., Satoh T., Koka M., Yokoyama A. (2015). Co-localization of iron binding on silica with p62/sequestosome1 (SQSTM1) in lung granulomas of mice with acute silicosis. J. Clin. Biochem. Nutr..

[44] Wang L., Li S., Yao Y., Yin W., Ye T. (2021). The role of natural products in the prevention and treatment of pulmonary fibrosis: A review. Food Funct..

[45] Tan S., Yang S., Chen G., Zhu L., Sun Z., Chen S. (2020). Trehalose alleviates apoptosis by protecting the autophagy-lysosomal system in alveolar macrophages during human silicosis. Life Sci..

[46] Liu H., Yu H., Cao Z., Gu J., Pei L., Jia M., Su M. (2019). Kaempferol modulates autophagy and alleviates silica-induced pulmonary fibrosis. DNA Cell Biol..

[47] Hu S., Zhao H., Al-Humadi N.H., Yin X.J., Ma J.K. (2006). Silica-induced apoptosis in alveolar macrophages: Evidence of in vivo thiol depletion and the activation of mitochondrial pathway. J. Toxicol. Environ. Health Part A.

[48] Bergsbaken T., Fink S.L., Cookson B.T. (2009). Pyroptosis: Host cell death and inflammation. Nat. Rev. Microbiol..

[49] Shi J., Gao W., Shao F. (2017). Pyroptosis: Gasdermin-mediated programmed necrotic cell death. Trends Biochem. Sci..

[50] Shi J., Zhao Y., Wang K., Shi X., Wang Y., Huang H., Zhuang Y., Cai T., Wang F., Shao F. (2015). Cleavage of GSDMD by inflammatory caspases determines pyroptotic cell death. Nature.

[51] Cassel S.L., Eisenbarth S.C., Iyer S.S., Sadler J.J., Colegio O.R., Tephly L.A., Carter A.B., Rothman P.B., Flavell R.A., Sutterwala F.S. (2008). The Nalp3 inflammasome is essential for the development of silicosis. Proc. Natl. Acad. Sci. USA.

[52] Yang J., Liu X., Bhalla K., Kim C.N., Ibrado A.M., Cai J., Peng T.I., Jones D.P., Wang X. (1997). Prevention of apoptosis by Bcl-2: Release of cytochrome c from mitochondria blocked. Science.

[53] Kluck R.M., Bossy-Wetzel E., Green D.R., Newmeyer D.D. (1997). The release of cytochrome c from mitochondria: A primary site for Bcl-2 regulation of apoptosis. Science.

[54] Fazzi F., Njah J., Di Giuseppe M., Winnica D.E., Go K., Sala E., St Croix C.M., Watkins S.C., Tyurin V.A., Phinney D.G. (2014). TNFR1/phox interaction and TNFR1 mitochondrial translocation Thwart silica-induced pulmonary fibrosis. J. Immunol..

[55] He X., Chen S., Li C., Ban J., Wei Y., He Y., Liu F., Chen Y., Chen J. (2020). Trehalose alleviates crystalline silica-induced pulmonary fibrosis via activation of the TFEB-mediated autophagy-lysosomal system in alveolar macrophages. Cells.

[56] Li B., Zeng M., He W., Huang X., Luo L., Zhang H., Deng D.Y. (2015). Ghrelin protects alveolar macrophages against lipopolysaccharide-induced apoptosis through growth hormone secretagogue receptor 1a-dependent c-Jun N-terminal kinase and Wnt/β-catenin signaling and suppresses lung inflammation. Endocrinology.

[57] Thibodeau M., Giardina C., Hubbard A.K. (2003). Silica-induced caspase activation in mouse alveolar macrophages is dependent upon mitochondrial integrity and aspartic proteolysis. Toxicol. Sci..

[58] Tan S., Yang S., Chen M., Wang Y., Zhu L., Sun Z., Chen S. (2020). Lipopolysaccharides promote pulmonary fibrosis in silicosis through the aggravation of apoptosis and inflammation in alveolar macrophages. Open Life Sci..

[59] Zhang L., He Y.L., Li Q.Z., Hao X.H., Zhang Z.F., Yuan J.X., Bai Y.P., Jin Y.L., Liu N., Chen G. (2014). N-acetylcysteine alleviated silica-induced lung fibrosis in rats by down-regulation of ROS and mitochondrial apoptosis signaling. Toxicol. Mech. Methods.

[60] Nagata S. (1996). Apoptosis mediated by the Fas system. Apostosis.

[61] Ware C.F., VanArsdale S., VanArsdale T.L. (1996). Apoptosis mediated by the TNF-related cytokine and receptor families. J. Cell. Biochem..

[62] Yao S.Q., Rojanasakul L.W., Chen Z.Y., Xu Y.J., Bai Y.P., Chen G., Zhang X.Y., Zhang C.M., Yu Y.Q., Shen F.H. (2011). Fas/FasL pathway-mediated alveolar macrophage apoptosis involved in human silicosis. Apoptosis.

[63] Hamzaoui A., Ammar J., Graïri H., Hamzaoui K. (2003). Expression of Fas antigen and Fas ligand in bronchoalveolar lavage from silicosis patients. Mediat. Inflamm..

[64] Borges V.M., Falcão H., Leite-Júnior J.H., Alvim L., Teixeira G.P., Russo M., Nóbrega A.F., Lopes M.F., Rocco P.M., Davidson W.F. (2001). Fas ligand triggers pulmonary silicosis. J. Exp. Med..

[65] Luo C., Ji X., Fan J., Hou Z., Wang T., Wu B., Ni C. (2016). Annexin A5 promotes macrophage activation and contributes to pulmonary fibrosis induced by silica particles. Toxicol. Ind. Health.

[66] Wang W., Liu H., Dai X., Fang S., Wang X., Zhang Y., Yao H., Zhang X., Chao J. (2015). p53/PUMA expression in human pulmonary fibroblasts mediates cell activation and migration in silicosis. Sci. Rep..

[67] Wang L., Bowman L., Lu Y., Rojanasakul Y., Mercer R.R., Castranova V., Ding M. (2005). Essential role of p53 in silica-induced apoptosis. Am. J. Physiol. Lung Cell. Mol. Physiol..

[68] Gouda M.M., Bhandary Y.P. (2019). Acute lung injury: IL-17A-mediated inflammatory pathway and its regulation by curcumin. Inflammation.

[69] Gouda M.M., Bhandary Y.P. (2018). Curcumin down-regulates IL-17A mediated p53-fibrinolytic system in bleomycin induced acute lung injury in vivo. J. Cell. Biochem..

[70] Bhandary Y.P., Shetty S.K., Marudamuthu A.S., Fu J., Pinson B.M., Levin J., Shetty S. (2015). Role of p53-fibrinolytic system cross-talk in the regulation of quartz-induced lung injury. Toxicol. Appl. Pharmacol..

[71] Bhandary Y.P., Shetty S.K., Marudamuthu A.S., Ji H.L., Neuenschwander P.F., Boggaram V., Morris G.F., Fu J., Idell S., Shetty S. (2013). Regulation of lung injury and fibrosis by p53-mediated changes in urokinase and plasminogen activator inhibitor-1. Am. J. Pathol..

[72] Cheng Y., Luo W., Li Z., Cao M., Zhu Z., Han C., Dai X., Zhang W., Wang J., Yao H. (2019). CircRNA-012091/PPP1R13B-mediated lung fibrotic Response in silicosis via endoplasmic reticulum stress and autophagy. Am. J. Respir. Cell Mol. Biol..

[73] Chang C.Y., Pan P.H., Wu C.C., Liao S.L., Chen W.Y., Kuan Y.H., Wang W.Y., Chen C.J. (2021). Endoplasmic reticulum stress contributes to gefitinib-induced apoptosis in glioma. Int. J. Mol. Sci..

[74] Zhang L., Xu D., Li Q., Yang Y., Xu H., Wei Z., Wang R., Zhang W., Liu Y., Geng Y. (2018). N-acetyl-seryl-aspartyl-lysyl-proline (Ac-SDKP) attenuates silicotic fibrosis by suppressing apoptosis of alveolar type II epithelial cells via mediation of endoplasmic reticulum stress. Toxicol. Appl. Pharmacol..

[75] Jiang P.R., Cao Z., Qiu Z.L., Pan J.W., Zhang N., Wu Y.F. (2015). Plasma levels of TNF-α and MMP-9 in patients with silicosis. Eur. Rev. Med. Pharmacol. Sci..

[76] Piguet P.F., Collart M.A., Grau G.E., Sappino A.P., Vassalli P. (1990). Requirement of tumour necrosis factor for development of silica-induced pulmonary fibrosis. Nature.

[77] Piguet P.F., Vesin C. (1994). Treatment by human recombinant soluble TNF receptor of pulmonary fibrosis induced by bleomycin or silica in mice. Eur. Respir. J..

[78] Gao H.S., Rong X., Peng D., Chen N.F., Bing M., Zhao H.B., Zhen Y., Wang S.X. (2011). Cross-talk of the related bioactivity mediators in serum after injection of soluble TNF-α receptor on silicosis model of rats. Toxicol. Ind. Health.

[79] Gambelli F., Di P., Niu X., Friedman M., Hammond T., Riches D.W., Ortiz L.A. (2004). Phosphorylation of tumor necrosis factor receptor 1 (p55) protects macrophages from silica-induced apoptosis. J. Biol. Chem..

[80] Gozal E., Ortiz L.A., Zou X., Burow M.E., Lasky J.A., Friedman M. (2002). Silica-induced apoptosis in murine macrophage: Involvement of tumor necrosis factor-α and nuclear factor-kB activation. Am. J. Respir. Cell Mol. Biol..

[81] Srivastava K.D., Rom W.N., Jagirdar J., Yie T.A., Gordon T., Tchou-Wong K.M. (2002). Crucial role of interleukin-1β and nitric oxide synthase in silica-induced inflammation and apoptosis in mice. Am. J. Respir. Crit. Care Med..

[82] Miwa K., Asano M., Horai R., Iwakura Y., Nagata S., Suda T. (1998). Caspase 1-independent IL-1β release and inflammation induced by the apoptosis inducer Fas ligand. Nat. Med..

[83] Yao S.Q., He Q.C., Yuan J.X., Chen J., Chen G., Lu Y., Bai Y.P., Zhang C.M., Yuan Y., Xu Y.J. (2013). Role of Fas/FasL pathway-mediated alveolar macrophages releasing inflammatory cytokines in human silicosis. Biomed. Environ. Sci..

[84] Song Z.S., Shao H., Chen Y.Q., Zhang R. (2018). Expression and significance of NLRP3/IL-1β/TGF-β(1) signal axis in rat model of silicosis pulmonary fibrosis. Zhonghua Lao Dong Wei Sheng Zhi Ye Bing Za Zhi.

[85] Li X., Yan X., Wang Y., Wang J., Zhou F., Wang H., Xie W., Kong H. (2018). NLRP3 inflammasome inhibition attenuates silica-induced epithelial to mesenchymal transition (EMT) in human bronchial epithelial cells. Exp. Cell Res..

[86] Song Z.S., Zhang R., Zhang J., Shao H. (2020). Inhibition of NLRP3 inflammasome activation on the inflammatory response of macrophage induced by silica dust. Zhonghua Lao Dong Wei Sheng Zhi Ye Bing Za Zhi.

[87] Reisetter A.C., Stebounova L.V., Baltrusaitis J., Powers L., Gupta A., Grassian V.H., Monick M.M. (2011). Induction of inflammasome-dependent pyroptosis by carbon black nanoparticles. J. Biol. Chem..

[88] Evavold C.L., Ruan J., Tan Y., Xia S., Wu H., Kagan J.C. (2018). The pore-forming Protein gasdermin D regulates interleukin-1 secretion from living macrophages. Immunity.

[89] Zhang E., Yang Y., Chen S., Peng C., Lavin M.F., Yeo A.J., Li C., Liu X., Guan Y., Du X. (2018). Bone marrow mesenchymal stromal cells attenuate silica-induced pulmonary fibrosis potentially by attenuating Wnt/β-catenin signaling in rats. Stem. Cell. Res. Ther..

[90] Li X., An G., Wang Y., Liang D., Zhu Z., Tian L. (2018). Targeted migration of bone marrow mesenchymal stem cells inhibits silica-induced pulmonary fibrosis in rats. Stem Cell. Res. Ther..

[91] Li X., Wang Y., An G., Liang D., Zhu Z., Lian X., Niu P., Guo C., Tian L. (2017). Bone marrow mesenchymal stem cells attenuate silica-induced pulmonary fibrosis via paracrine mechanisms. Toxicol. Lett..

[92] Zhao Q., Hao C., Wei J., Huang R., Li C., Yao W. (2021). Bone marrow-derived mesenchymal stem cells attenuate silica-induced pulmonary fibrosis by inhibiting apoptosis and pyroptosis but not autophagy in rats. Ecotoxicol. Environ. Saf..

[93] Zhu H.X., Gao J.L., Zhao M.M., Li R., Tian Y.X., Wang X., Zhang J., Yuan J.X., Cui J.Z. (2016). Effects of bone marrow-derived mesenchymal stem cells on the autophagic activity of alveolar macrophages in a rat model of silicosis. Exp. Ther. Med..

[94] Wierenga K.A., Wee J., Gilley K.N., Rajasinghe L.D., Bates M.A., Gavrilin M.A., Holian A., Pestka J.J. (2019). Docosahexaenoic acid suppresses silica-induced inflammasome activation and IL-1 cytokine release by interfering with priming signal. Front. Immunol..

[95] Rajasinghe L.D., Chauhan P.S., Wierenga K.A., Evered A.O., Harris S.N., Bates M.A., Gavrilin M.A., Pestka J.J. (2020). Omega-3 docosahexaenoic acid (DHA) impedes silica-induced macrophage corpse accumulation by attenuating cell death and potentiating efferocytosis. Front. Immunol..

[96] Eisenberg-Lerner A., Bialik S., Simon H.U., Kimchi A. (2009). Life and death partners: Apoptosis, autophagy and the cross-talk between them. Cell Death Differ..

[97] Maiuri M.C., Le Toumelin G., Criollo A., Rain J.C., Gautier F., Juin P., Tasdemir E., Pierron G., Troulinaki K., Tavernarakis N. (2007). Functional and physical interaction between Bcl-X_L_ and a BH3-like domain in Beclin-1. EMBO J..

[98] Yang P., Song R., Li N., Sun K., Shi F., Liu H., Shen F., Jiang S., Zhang L., Jin Y. (2020). Silica dust exposure induces autophagy in alveolar macrophages through switching Beclin1 affinity from Bcl-2 to PIK3C3. Environ. Toxicol..

[99] Nakano K., Vousden K.H. (2001). PUMA, a novel proapoptotic gene, is induced by p53. Mol. Cell.

[100] Liu H., Cheng Y., Yang J., Wang W., Fang S., Zhang W., Han B., Zhou Z., Yao H., Chao J. (2017). BBC3 in macrophages promoted pulmonary fibrosis development through inducing autophagy during silicosis. Cell Death Dis..

[101] Chen H.P., Zhou Y., Qin X.F., Wang L., Lin X.F., Chen H., Hu Y.B. (2020). Endoplasmic reticulum stress regulates autophagy and tumor necrosis factor-α secretion of RAW264.7 cells induced by silica. Zhonghua Lao Dong Wei Sheng Zhi Ye Bing Za Zhi.

[102] Hornung V., Bauernfeind F., Halle A., Samstad E.O., Kono H., Rock K.L., Fitzgerald K.A., Latz E. (2008). Silica crystals and aluminum salts activate the NALP3 inflammasome through phagosomal destabilization. Nat. Immunol..

[103] Jessop F., Hamilton R.F., Rhoderick J.F., Shaw P.K., Holian A. (2016). Autophagy deficiency in macrophages enhances NLRP3 inflammasome activity and chronic lung disease following silica exposure. Toxicol. Appl. Pharmacol..

[104] Rogers C., Fernandes-Alnemri T., Mayes L., Alnemri D., Cingolani G., Alnemri E.S. (2017). Cleavage of DFNA5 by caspase-3 during apoptosis mediates progression to secondary necrotic/pyroptotic cell death. Nat. Commun..

[105] Wang Y., Gao W., Shi X., Ding J., Liu W., He H., Wang K., Shao F. (2017). Chemotherapy drugs induce pyroptosis through caspase-3 cleavage of a gasdermin. Nature.

